# Effects of alkali contamination on mechanical properties and microstructure of red clay

**DOI:** 10.1038/s41598-026-37873-5

**Published:** 2026-01-30

**Authors:** Lianrui Wang, Jun Chen, Dongdong Liu, Yujie Lan

**Affiliations:** 1 College of Resources and Environmental Engineering, Guizhou University, Guiyang, 550025 China; 2 Guizhou Institute of Water Resources Science, Guiyang, 550002 China; 3 Guizhou Institute of Technology, Guiyang, 550007 China

**Keywords:** Red clay, Alkali contamination, Mechanical properties, Microstructure, Concentration effect, Engineering, Environmental sciences, Materials science

## Abstract

Alkaline environments significantly affect the engineering properties of red clay. Although the effects of alkali contamination on the microstructure and mechanical characteristics of cohesive soils have been extensively studied, systematic research focusing on red clay remains limited. This study employed NaOH solution to simulate alkali contamination of red clay. By utilizing unconsolidated undrained (UU) triaxial shear tests, mercury intrusion porosimetry (MIP), scanning electron microscopy (SEM), laser particle size analysis (LPSA), and X-ray diffraction (XRD), this work systematically investigated the influence of different alkali concentrations on the mechanical properties and microstructure of red clay. The results indicate a distinct threshold effect of alkali concentration on red clay behavior, with the existence of a most unfavorable concentration (3.5%) and an optimal concentration (14%). At the 3.5% concentration, destructive dissolution was dominant, in which mineral dissolution led to particle refinement, surface smoothing, and an increase in the proportion of large pores, resulting in a significant reduction in soil strength. At 14% concentration, reconstructive cementation became prevalent. XRD analysis confirmed the formation of new crystalline phases (sodium aluminosilicate), indicating the occurrence of geopolymerization. The newly formed cementitious materials effectively bonded soil particles and filled pores, thereby enhancing shear strength, although their brittle nature resulted in strain softening during shearing. At excessively high concentrations (e.g., 21%), structural degradation and strength reduction were again observed. This research reveals the dynamic evolution of the “dissolution-cementation” competition mechanism in red clay under different concentrations of alkali contamination, providing a theoretical basis for preventing alkali contamination in red clay foundations and for soil reinforcement techniques based on alkali activation principles.

## Introduction

In recent decades, rapid urbanization and industrialization have led to severe soil contamination due to the large-scale leakage and infiltration of industrial effluents and domestic sewage into subsurface environments^1,2^. It is well established that soil pollution alters the mechanical properties and microstructure of soils, thereby affecting the stability and safety of overlying structures^3–5^. Among various types of contaminants, alkali contamination represents a significant category. Alkali solution leakage is common in industries such as paper manufacturing, textiles, aluminum production, and paint manufacturing^6^. Kabanov et al.^7^ reported that in Uralsk, extensive leakage of alkali solutions (e.g., NaOH) from aluminum plants caused the swelling of foundation soils, leading to structural deformation in buildings. Sinha et al.^8^ discovered that the bearing capacity of foundation soils in an aluminum plant diminished by about 33% following contamination from leaked caustic soda solution. Similarly, Wang et al.^9^ documented a case in Guizhou, China, where the infiltration of alkali solution significantly altered the physico-mechanical properties and microstructure of the red clay foundation soils at an aluminum plant. Collectively, these cases demonstrate that alkali contamination-induced changes in soil engineering properties pose critical geotechnical challenges, compromising the structural safety of overlying infrastructure.

Existing studies have confirmed that alkali solutions can dissolve primary clay minerals, disrupt soil structure, and react to form new compounds, thereby altering soil mechanical properties and microstructure^10–15^. Elert et al.^16^ treated clays from the Alhambra Formation with three alkali solutions (Ca(OH)₂, NaOH, and KOH), revealing that all treatments initially caused the deterioration of expansive clay minerals (montmorillonite). Notably, NaOH and KOH solutions were more effective in transforming the clays into stable, albeit poorly crystalline, alkali aluminosilicates, calcium silicate hydrates, or interstratified illite-montmorillonite phases, which enhanced the soil’s structural stability. Sruthi and Reddy^17^ investigated the effects of alkali solutions with varying interaction volumes on the microstructural properties and mineralogical alterations of kaolinitic clays. Their study demonstrated that red earth and kaolin treated with alkali solutions (4 N NaOH and 4 N KOH) at 110 °C exhibited complete mineral dissolution at higher interaction volumes. Subsequently, Sruthi and Reddy^18^ further examined the swelling behavior and mineralogical characteristics of alkali-transformed (partially or fully transformed) kaolinitic clays. Their findings revealed that the swelling behavior was directly correlated with the formation of neo-formed minerals and the dissolution of primary minerals. Meanwhile, Emmanuel et al.^19^ demonstrated that a two-step activation process, which combines NaOH treatment followed by sodium silicate solution activation, significantly enhances the reactivity of kaolinite, generating substantial amounts of highly depolymerized gels. This reaction mechanism has been shown to effectively enhance the mechanical properties of kaolinite-rich clays. Yue et al.^20^ systematically investigated the effects of different concentrations of alkali solution (NaOH) on the mechanical properties and microstructure of lateritic soil through unconfined compressive strength tests, mercury intrusion porosimetry (MIP), nitrogen adsorption tests, and other methods. The results showed that alkali erosion leads to the formation of aluminosilicate gels, which can fill soil pores and cement soil particles. However, in highly alkaline environments (pH ≥ 12.4), gel formation is inhibited and the soil skeleton is degraded, resulting in reduced strength. Barakat et al.^21^ conducted experiments using highly alkaline solutions that simulated the chemical composition and pH of concrete pore water to treat the Opalinus Clay from the lower sandy facies of the Mont Terri site, Switzerland. The study revealed that the highly alkaline solutions significantly altered the clay’s water retention characteristics and microstructure through acid-base reactions, mineral dissolution, and structural dispersion. These processes led to increased porosity, reduced suction, and a potential increase in permeability.

In summary, the interaction between alkali solutions and clay minerals exhibits a marked duality. On the one hand, alkali solutions can dissolve mineral components and disrupt soil structure, leading to the degradation of engineering properties^22,23^. On the other hand, alkali activation can promote the formation of cementitious materials, thereby improving soil performance^24^. This dual nature means that alkali solutions can be viewed both as a contaminant and as a potential stabilizing agent in geotechnical engineering. Although alkali-activated stabilization techniques have been applied in collapsible loess^25^, systematic research on typical cohesive soils such as red clay remains relatively limited. Similarly, microbially induced calcium carbonate precipitation (MICP) is another soil improvement method based on a cementation mechanism, yet the two differ significantly in their mechanisms and performance characteristics. MICP technology utilizes urease-producing bacteria to metabolically generate carbonate ions, which combine with calcium ions in the environment to form calcium carbonate crystals^26^. It offers advantages such as environmental friendliness, minimal construction disturbance, and stable cementation products^27^. However, it also faces challenges including higher costs, difficulty in controlling the uniformity of cementation, and poor grouting efficiency in low-permeability clays. Currently, MICP is mainly used for sand reinforcement^28–31^. In contrast, alkali-activated stabilization directly relies on the dissolution of aluminosilicate phases in clay minerals and subsequent cementitious reactions. It features lower cost and rapid reaction but is highly sensitive to alkali concentration, exhibiting a clear “concentration threshold” phenomenon^32,33^. Therefore, systematically clarifying the influence of alkali concentration on the mechanical properties and microstructure of red clay is not only crucial for assessing the engineering risks of alkali pollution but also provides key scientific insights for optimizing the application of alkali-activated stabilization techniques in cohesive soils.

Red clay, a type of highly plastic clay formed through intense chemical weathering of carbonate rocks, typically exhibits hues of brownish-red, reddish-brown, or yellowish-brown^34–36^. China possesses one of the world’s most extensive distributions of red clay, primarily concentrated in regions south of the Yangtze River^37–39^. This clay contains minerals such as kaolinite and quartz that readily interact with alkaline solutions, making it particularly susceptible to property alterations when exposed to alkaline contaminants in natural environments. However, current research on alkali-contaminated red clay remains limited, with existing studies being relatively fragmented and often presenting contradictory conclusions. To address this research gap, this study employs red clay as the experimental subject. Using NaOH solutions at varying concentrations, a comprehensive experimental approach—including unconsolidated undrained triaxial shear tests (UU), MIP, scanning electron microscopy (SEM), laser particle size analysis (LPSA), and X-ray diffraction (XRD)—was adopted to systematically elucidate the influence of different alkali contamination levels on the mechanical properties and microstructural characteristics of red clay.

## Materials and methodology

### Material studied

**Fig. 1:**
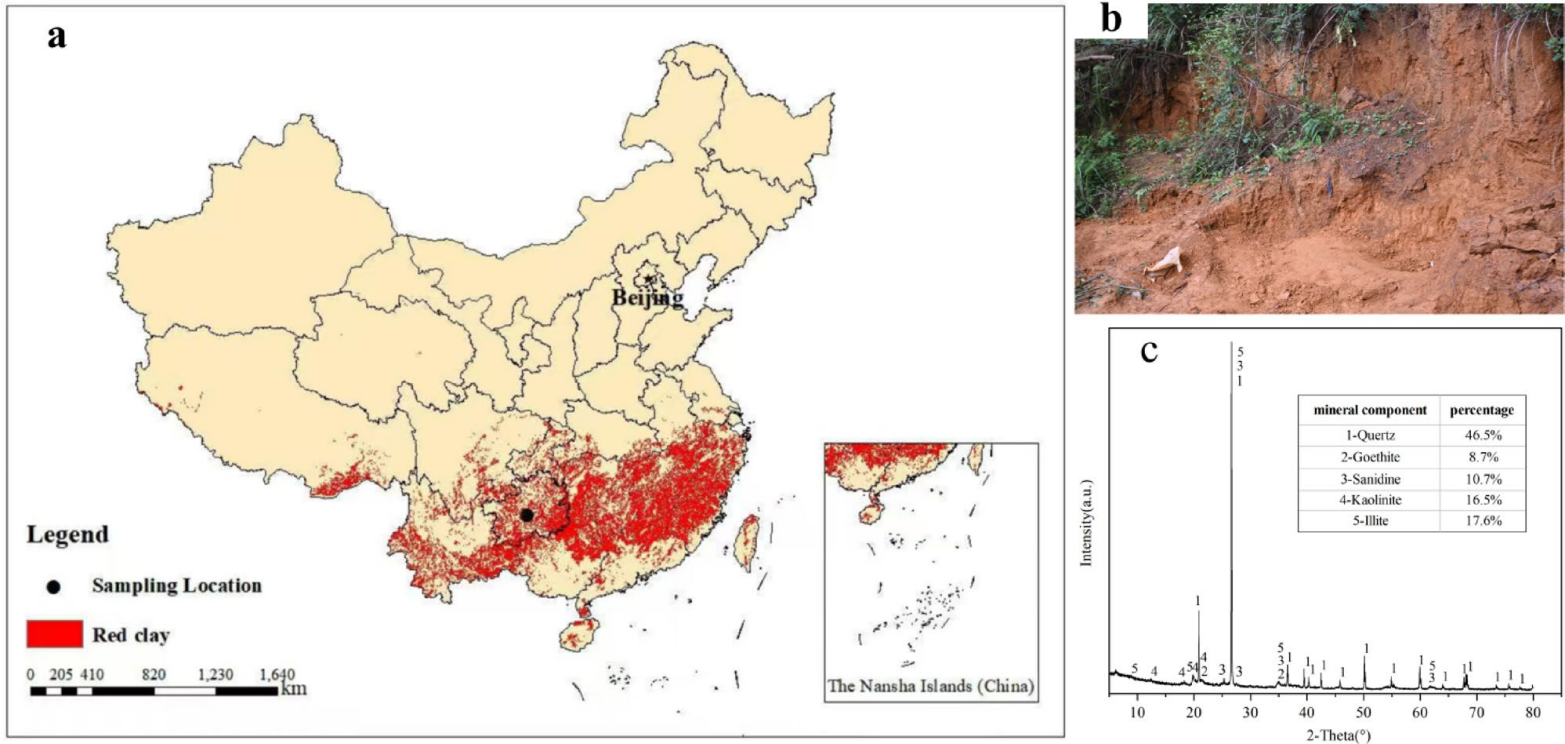
Basic characteristics of the red clay. (**a**) Distribution of red clay in China and sampling locations (map generated using ArcMap 10.7, Esri, Redlands, CA, USA; https://www.esri.com). (**b**) The red clay used in the experiments. (**c**) The XRD test results of red clay.

The sampling site is situated at the geophysical prospecting test site on the West Campus of Guizhou University, Guizhou Province, China (26°26’38.40"N, 106°39’30.16"E; Fig. 1a). The collected red clay exhibits a yellowish-brown color and is primarily in a plastic to semi-solid state (Fig. 1b). XRD analysis reveals that its mineral composition is dominated by quartz, with subsidiary goethite, sanidine, kaolinite, and illite (Fig. 1c). Following the Chinese national standard Standard for *Geotechnical Testing Method* (GB/T 50123 − 2019), the basic physical properties of the red clay were determined; the results are summarized in Table 1.

**Table 1:** Main physical parameters of red clay.

Natural density (g·cm^− 3^)	Natural moisture content w (%)	Specific gravity G_s_	Plastic limit W_*p*_ (%)	Liquid limit W_L_ (%)	Plasticity index I_*p*_ (%)	Optimum moisture content w (%)	Maximum dry density (g·cm^− 3^)
1.734	35.12	2.65	30.47	50.13	19.66	26	1.576

### Sample preparation

Given the proven high reactivity between NaOH solutions and clay minerals^40^, this study employed NaOH to prepare alkali solutions at varying concentrations (where concentration refers to the mass percentage of NaOH in the solution). Preliminary studies indicated that curing under optimum moisture content for 10 days induced the most significant alterations in the properties of red clay^41^. To investigate the underlying mechanisms, specimens were prepared at the determined optimum water content of 26%. During preparation, the alkali solution was used as the sole liquid phase to achieve the target moisture content, with no additional water introduced.

To better simulate real conditions, actual wastewater and the highest-concentration feed liquor from an alumina plant were analyzed to design the alkali concentrations. Based on the calculation that all Na⁺ was provided by NaOH, the wastewater was equivalent to a NaOH solution with a concentration of 0.7% to 3.5%, while the feed liquor corresponded to a NaOH concentration of 22.5%. Accordingly, six concentration levels—0% (plain soil), 0.7%, 3.5%, 7%, 14%, and 21%—were established for testing. Deionized water was used as the solvent to exclude interference from external ions.

**Fig. 2:**
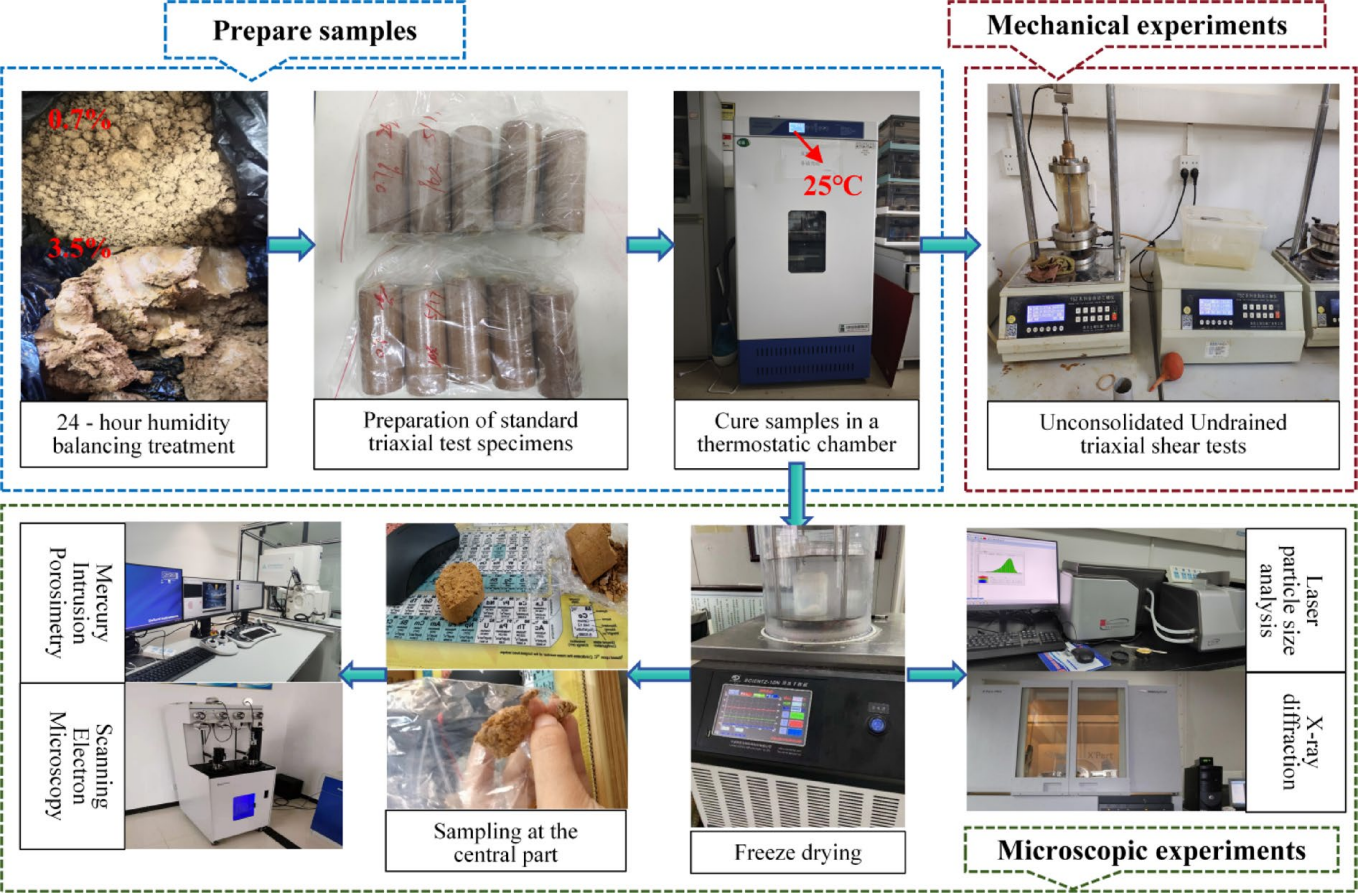
Sample preparation and experimental procedures.

Figure 2 schematically outlines the sample preparation and testing procedures. Air-dried red clay was pulverized, sieved (≤ 2 mm), and homogenized with designated alkali solutions. After 24 h of homogenization, triaxial specimens (39.1 mm in diameter × 80 mm in height) were prepared according to a unified standard and cured at 25 °C for 10 days. Mechanical tests were conducted in triplicate to ensure reliability. To preserve the integrity of the microstructure, specimens intended for microstructural characterization were treated using the freeze-drying method—a technique superior to oven- or air-drying in preventing pore collapse^42^. Undisturbed cured specimens were directly subjected to freeze-drying to minimize structural disturbance. After drying, the central portion of each specimen was extracted for subsequent analysis to avoid edge effects.

## Experimental methods

### Mechanical testing

UU tests were conducted in strict compliance with the Chinese national standard *Standard for geotechnical testing methods* (GB/T 50123 − 2019). A conventional triaxial apparatus was then employed to perform UU tests on alkali-contaminated samples at varying concentrations. Tests were performed under confining pressures of 100, 200, and 300 kPa, with axial shearing applied at a strain rate of 0.66 mm/min until an axial strain of 18% was reached. Deviatoric stress (${\sigma _1} - {\sigma _3}$) versus axial strain (${\varepsilon _1}$) relationships (stress-strain curves) under different confining pressures were derived from experimental data. For curves exhibiting peak stress, the maximum value was adopted as the shear strength; for non-peaking responses, the stress at 15% axial strain was designated as shear strength^43^. Strength envelopes were subsequently constructed using Mohr’s circle analysis, enabling calculation of shear strength parameters (cohesion and friction angle) for each specimen.

To evaluate the statistical impact of alkali solution concentration on shear strength, and given the well-established influence of confining pressure on soil strength, data analysis was performed separately for each confining pressure group (100, 200, and 300 kPa). At each confining pressure level, a one-way analysis of variance (One-way ANOVA) was conducted to examine differences in shear strength among the groups with different alkali concentrations. If the ANOVA results indicated a statistically significant difference (*p* < 0.05), the Tukey’s HSD post-hoc test was subsequently applied for multiple comparisons to identify which specific concentration groups differed significantly. All analyses were performed using SPSS, with the significance level set at α = 0.05.

### MIP

MIP is a widely employed technique for characterizing soil pore structures, particularly for pore size analysis^44–46^. This experimental investigation was conducted in accordance with the Chinese National Standard Determination of *Pore Size Distribution and Porosity of Solid Materials by Mercury Porosimetry and Gas Adsorption* (GB/T 21650). Measurements were performed using a Micromeritics AutoPore IV 9600 high-performance automated mercury porosimeter with an operational range of 0.005–340 μm. For pore size calculations in this study, a mercury contact angle ($\theta$) of 130° and a mercury surface tension (${\sigma _{H{\mathrm{g}}}}$) of 0.485 N/m were adopted.

### Fractal dimension principle

Fractal dimension serves as a robust indicator for characterizing the complexity of soil pore structures. Fractal models based on MIP data analysis include the space-filling model, menger sponge model, and thermodynamics-based fractal models such as the Zhang and Li model^47^. Compared with other fractal models, the reliability and accuracy of the Zhang and Li model have been extensively validated^48–50^. In this experimental study, the Zhang and Li model was employed to analyze pore structure complexity in alkali-contaminated red clay. Based on the fundamental mathematical definition, the energy increase at pore surfaces equals the work performed by external atmospheric pressure on mercury^51^, with its integral equation expressed as:

1 $$\int_{0}^{V} {P{\mathrm{dV=}} - \int_{0}^{s} {\sigma \cos \theta ds} }$$

where *S* is the pore surface area of the specimen ${\mathrm{(}}{{\mathrm{m}}^2})$.

The surface tension $\sigma$ is a constant in the test, and the fractal dimension *D* of the pore distribution is used to represent the link between the pore diameter *r* and the incoming mercury volume *V*. Equation (1) is written as a discrete model:

2 $$\sum\limits_{{i=1}}^{n} {{{\bar {P}}_i}\Delta {V_i}} =Cr_{n}^{{2 - D}}V_{n}^{{\frac{D}{3}}}$$

where $\bar {P}$ is the average of the external pressure applied during the first injection $(Pa)$; $\Delta {V_i}$ is the amount of mercury fed during the ${i_{th}}$ injection ${\mathrm{(}}{{\mathrm{m}}^3})$; *n* is the number of mercury injection pressure intervals; ${r_n}$ is the pore radius corresponding to the ${n_{th}}$ injection; ${V_n}$ is the total volume of *n* injections; *D* is the pore fractal dimension; and *C* is the coefficient.

Make

3 $${W_n}=\sum\limits_{{i=1}}^{n} {{{\bar {P}}_i}\Delta {V_i}}$$

4 $${Q_n}=V_{n}^{{{\raise0.7ex\hbox{$1$} \!\mathord{\left/ {\vphantom {1 3}}\right.\kern-0pt}\!\lower0.7ex\hbox{$3$}}}}{r_n}$$

Combining Eqs. (3) and (4) with Eq. (2) gives

5 $$\ln ({W_n}/r_{n}^{2})=D\ln {Q_n}+C$$

where $$C=\ln C$$.

A relationship curve was plotted with $\ln {Q_n}$ as the abscissa and $\ln ({W_n}/r_{n}^{2})$ as the ordinate. Linear fitting was applied to this curve, and the resulting D-value (slope) was adopted as the fractal dimension of soil pores. The D-value typically ranges between 2 and 3: when D approaches 2, pore walls tend toward smoothness; when D approaches 3, pore walls exhibit increased roughness characterized by micro-cracks, irregular morphologies, and protuberant structures.

### LPSA

The particle size distribution of the soil samples was measured using a Bettersize 3000Plus laser particle size analyzer (Bettersize Instruments Ltd., China), which has a detection range of 0.02–3500 μm and a repeatability error of ≤ 0.5%. Due to the natural dispersing effect of sodium hydroxide solution, laser particle size analysis (LPSA) was performed without sample pretreatment to investigate the influence of alkali contamination at different concentrations on particle size characteristics. The dried specimens were ground and sieved through a 2 mm mesh before analysis. Prior to measurement, the soil suspension in the sample cell was ultrasonically dispersed for 5 min, and duplicate measurements were taken to ensure data reliability.

### SEM

SEM observations were conducted using an FEI/INSPECT F50 microscope (USA) to examine particle morphology and arrangement. Prior to SEM analysis, samples were prepared by: (1) creating a 1 mm-deep notch at the center; (2) fracturing along the notch to expose fresh surfaces; (3) removing loose particles with compressed air; and (4) selecting representative areas for gold sputter-coating.

### XRD

XRD analysis was performed using a PANalytical X’Pert PRO MPD diffractometer (PANalytical B.V., the Netherlands) to identify changes in the mineral and crystalline phases of the soil samples. This analysis provides direct mineralogical evidence for the formation of new cementitious phases during the alkali-soil reaction. The tests were conducted over a 2$\theta$ range of 5° to 80° in continuous scan mode at a scanning speed of 10°/min and a temperature of 20 °C. The obtained data were processed and analyzed using the X’Pert HighScore Plus software (Version 5.2; Malvern Panalytical B.V., Almere, The Netherlands; https://www.malvernpanalytical.com/en/products/product-range/highscore-plus-suite.html).

## Analysis of experimental results

### Analysis of mechanical property characteristics

#### Stress-strain curve

**Fig. 3:**
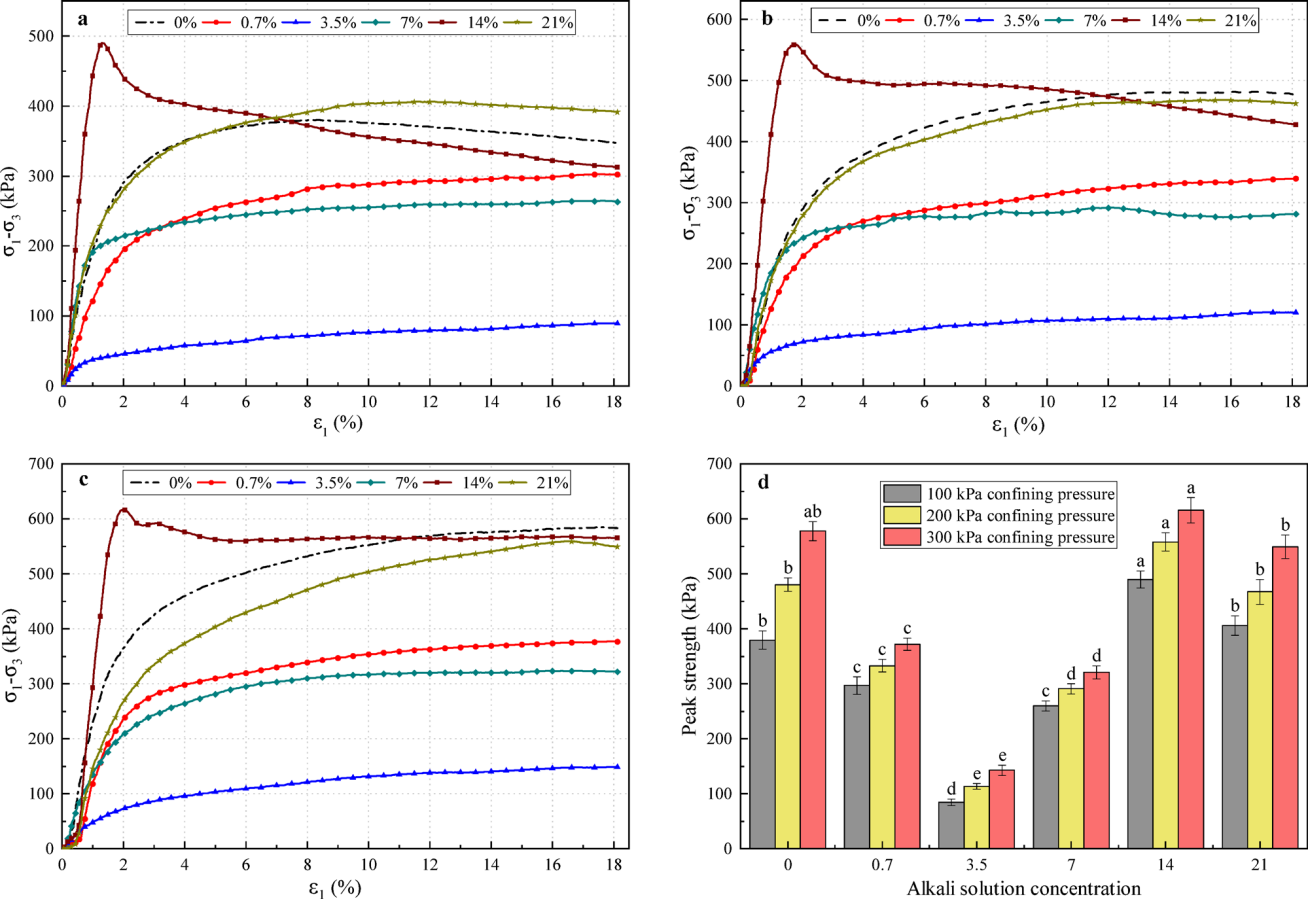
Mechanical test results of alkali-contaminated red clay at varying concentrations. (**a**) Stress-strain curves at 100 kPa. (**b**) Stress-strain curves at 200 kPa. (**c**) Stress-strain curves at 300 kPa. (**d**) Shear strength of alkali-contaminated red clay with different concentrations under various confining pressures. **Note**: ${\sigma _1} - {\sigma _3}$ denotes the deviatoric stress; ${\varepsilon _1}$ represents the axial strain.

Figure 3(a, b, c) presents stress-strain curves of alkali-contaminated red clay under three confining pressures at varying concentrations. As shown in Fig. 3(a, b, c), plain soil (0%) exhibits strain-softening behavior at 100 kPa confining pressure but demonstrates strain-hardening characteristics at 200 kPa and 300 kPa, consistent with typical properties of remolded red clay^52^. Under different confining pressures, as the concentration of the alkali solution increases, the evolution pattern of the stress-strain curves of the soil samples generally remains consistent. The measured shear strength first decreases, then increases, and subsequently decreases again.

Figure 3d summarizes the influence of alkali concentration on the peak shear strength under different confining pressures, along with the results of statistical significance analysis. When the concentration reached 0.7% and 3.5%, the shear strength of the soil samples decreased, with the lowest strength observed at 3.5%. At this concentration, the strength was significantly lower than that of all other concentrations under every confining pressure. The corresponding stress–strain curves exhibited strain‑hardening characteristics, indicating that within this concentration range the alkali solution acted primarily in a destructive manner, disrupting the soil structure, loosening particle arrangement, and thereby reducing strength. As the concentration increased to 7% and 14%, the shear strength began to recover, reaching its maximum at 14%. Under confining pressures of 100 kPa and 200 kPa, the strength at 14% was significantly higher than that of all other concentration groups. Under 300 kPa, it was significantly higher than the 0%, 0.7%, 3.5%, and 7% groups. Although the difference compared to the 21% group was not statistically significant at 300 kPa, the measured value was still numerically higher. The corresponding stress–strain curves shifted to strain‑softening behavior, displaying a distinct peak shear stress. This suggests that at this concentration, the alkali solution played a predominantly reinforcing role, leading to tighter particle arrangement and enhanced strength. When the concentration was further raised to 21%, the stress-strain curves resembled those of ordinary soil, and the shear strength decreased once again.

#### Shear strength index

**Fig. 4:**
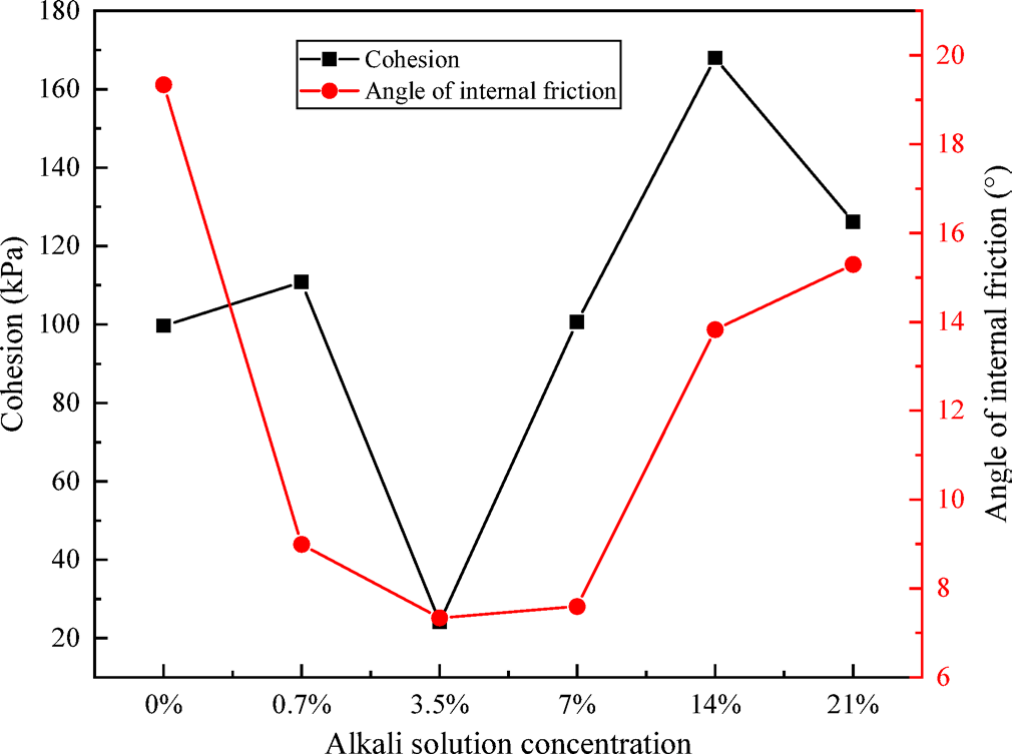
Shear strength parameters of red clay under alkali contamination at various concentrations.

The shear strength parameters were determined by calculating the average peak strengths obtained under different alkali concentrations and confining pressures. Figure 4 presents the shear strength parameters of all specimens. The plain soil exhibits cohesion (*c*) of 99.62 kPa and an internal friction angle ($\varphi$) of 19.34°. With increasing alkali concentration: *c* initially increases slightly, then decreases sharply to a minimum (24.1 kPa, 75.8% reduction) at 3.5% concentration. It subsequently peaks at 14% concentration (168 kPa, 68.6% increase) before decreasing to 126.2 kPa at 21% concentration (26.7% higher than plain soil); $\varphi$ decreases initially, reaching a minimum (7.34°, 62.0% reduction) at 3.5% concentration, then increases but remains below plain soil values at all concentrations.

Overall, at 3.5% concentration, both *c* and $\varphi$ reach minima, causing significant shear strength reduction. At 14% concentration, substantially increased *c* compensates for reduced $\varphi$, yielding higher shear strength than plain soil.

### Analysis of pore characteristics

#### Pore distribution characteristics

**Fig. 5:**
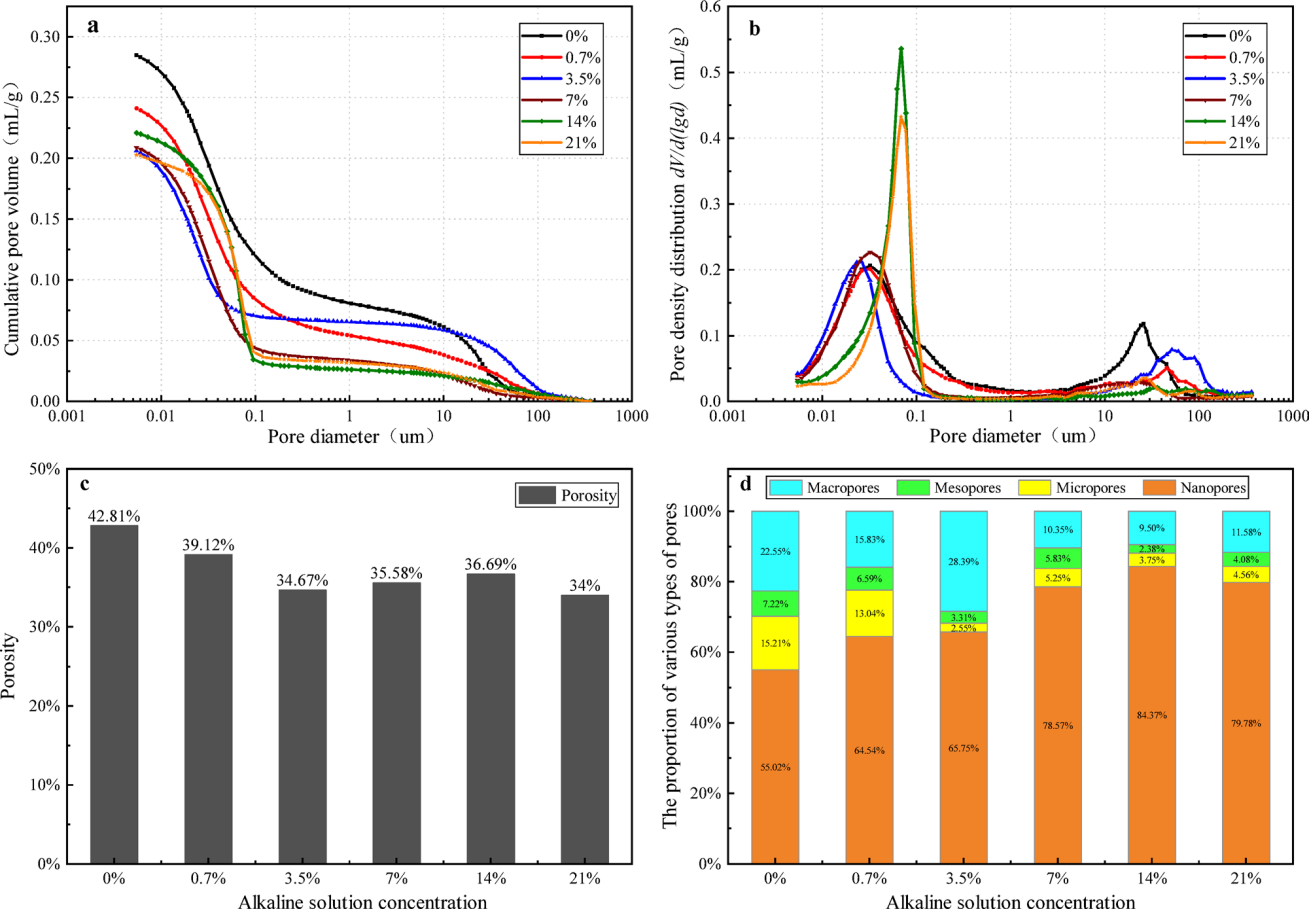
Pore structure characteristics of alkali-contaminated red clay. (**a**) Cumulative pore volume curve. (**b**) Log-differential mercury volume curves. (**c**) Porosity. (**d**) Proportion of various types of pores.

Figure 5a displays the cumulative pore volume curves for each soil sample, illustrating the variation of pore volume with pore diameter. Pores in the plain soil are continuously distributed across the entire pore size range. Based on the variation characteristics of cumulative pore volume curves in plain soils and with reference to previous studies on the pore structure of clay^53,54^, we classified soil pores into four main categories: macropores (d > 10 μm), mesopores (1 μm < d ≤ 10 μm), micropores (0.1 μm < d ≤ 1 μm), and nanopores (d ≤ 0.1 μm). Compared to the plain soil, the cumulative pore volumes of alkali-contaminated red clay samples are all reduced, with the most significant reduction observed in the 3.5% concentration sample.

Figure 5b presents the log differential mercury volume curves for each soil sample, reflecting the volume distribution characteristics of pores with different diameters. At alkali concentrations of 0%, 0.7%, and 3.5%, the curves exhibit a distinct bimodal structure, with peak fluctuation ranges primarily distributed within the macropore and nanopore regions. Notably, the macropore peak fluctuation range for the 3.5% concentration sample shifts towards larger pore diameters, indicating a significant increase in macropore size. In contrast, at concentrations of 7%, 14%, and 21%, the curves show only minor undulations without distinct peaks in the macropore region, exhibiting an overall unimodal morphology. This confirms that nanopores dominate the pore structure in these samples.

Figure 5c and d present variations in total porosity and pore size distribution of soil samples, respectively. Compared to plain soil, alkali-contaminated red clay exhibits reduced total porosity, indicating alkali-induced structural alterations that promote denser particle packing. At 3.5% concentration, porosity reaches its minimum (34.67%), yet macropore proportion peaks (28%). During preparation, samples at this concentration displayed soft, viscous behavior with high deformability, resembling silty clay. This elevated plasticity facilitated macropore formation during molding and curing. Combined with significant mechanical strength reduction, this suggests severe degradation of cementitious components within the clay matrix, destabilizing the soil structure.

Conversely, at the 14% alkali concentration, the proportion of nanopores significantly increases to 84.37%, while the macropore proportion drops to its minimum (9.5%). Concurrently, macroscopic mechanical properties show a substantial enhancement in shear strength. This indicates that the prolonged reaction between the alkali solution and red clay at this concentration generates substantial new-formed cementing substances that effectively infill soil pores and cement soil particles, thereby reinforcing the soil structure.

### Pore structural complexity

**Fig. 6:**
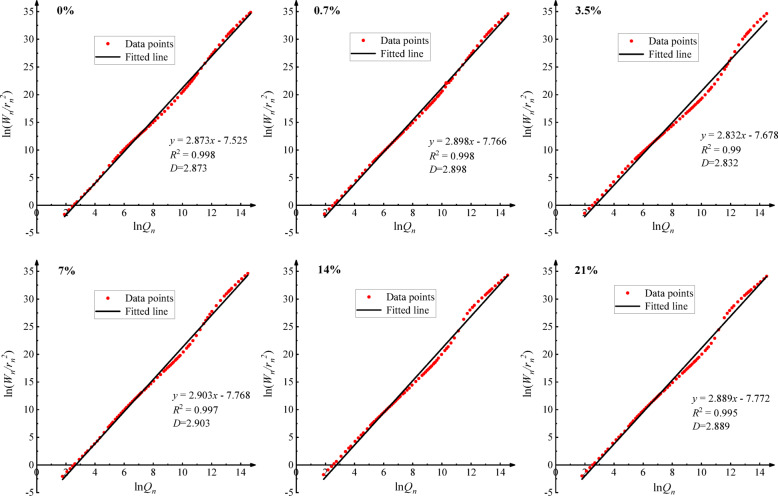
Fitted fractal dimension results of alkali-contaminated red clay samples.

Figure 6 presents the calculation results of pore fractal dimension (D) for each soil sample, characterizing the variation in roughness of pore walls in alkali-contaminated red clay. The data show excellent fitting results, with all correlation coefficients R² > 0.99. The D-values fall within the theoretical range of 2–3, indicating that the Zhang and Li model is suitable for quantitatively evaluating the roughness characteristics of red clay pore structure.

Data analysis reveals that alkali contamination significantly alters the micromorphology of pore walls in red clay and shows clear concentration dependence. Notably, the variation trend of pore roughness is significantly correlated with soil mechanical strength, with special responses observed at 3.5% and 14% contamination concentrations: at 3.5% concentration, the minimum D-value (2.832) indicates the smoothest pore walls, corresponding to the lowest mechanical strength; at 14% concentration, the maximum D-value (2.913) indicates the roughest pore walls, corresponding to the highest mechanical strength.

### Analysis of particle characteristics

#### Particle size characteristics

Table 2 presents the results of the particle size analysis for various soil samples. According to the Chinese National *Standard for engineering classification of soil* (GB/T 50145 − 2007), soil particles are classified into the following size ranges: clay particles (d ≤ 5 μm), silt particles (5 μm < d ≤ 75 μm), and sand particles (75 μm < d ≤ 2 mm). The red clay predominantly consists of clay and silt particles with relatively small sizes. The plain soil has a volume mean diameter of 13.89 μm and a peak particle size of 19.94 μm, with a clay particle content of 41.63%. The experimental results indicate that alkali contamination significantly impacts the particle size distribution of red clay, and these changes correlate clearly with the evolution of the soil’s mechanical strength. Under low alkali concentrations (0.7% and 3.5%), the dispersive and dissolving effects of the alkali solution cause the dissociation of some aggregates, resulting in particle refinement. Notably, the soil sample treated with a 3.5% alkali concentration displays the smallest particle size characteristics, with the volume mean diameter and peak particle size decreasing to 11.14 μm and 15.27 μm, respectively, while the clay particle content increases to 46.46%. As the alkali concentration rises to high levels (7% and 14%), the dispersed soil particles undergo re-cementation and form new aggregate structures. Particularly under the 14% alkali concentration, the particle composition of the soil sample undergoes a significant transformation: the volume mean diameter and peak particle size surge to 802.9 μm and 883.05 μm, respectively, while the clay particle and silt particle content decreases substantially, and the sand particle dominates, accounting for 91.15% of the composition. However, when the alkali concentration further increases to 21%, the aggregate structure begins to dissociate again, manifesting as a reduction in particle size, an increase in clay particle and silt particle content, and a corresponding decrease in the sand particle.

**Table 2:** Particle size analysis results of red clay subjected to alkali contamination at various concentrations.

Solution concentration	Volume average diameter (µm)	Peak particle size (µm)	Clay particle content (%)	Silt particle content (%)	Sand particle content (%)
0%	13.89	19.94	41.63	56.69	1.68
0.7%	11.32	16.11	43.68	55.07	1.25
3.5%	11.14	15.27	46.46	52.91	0.63
7%	12.94	18.43	37.51	61.56	0.93
14%	802.90	883.05	3.08	5.77	91.15
21%	404.90	876.55	22.39	31.68	45.93

#### Particle morphology characteristics

**Fig. 7:**
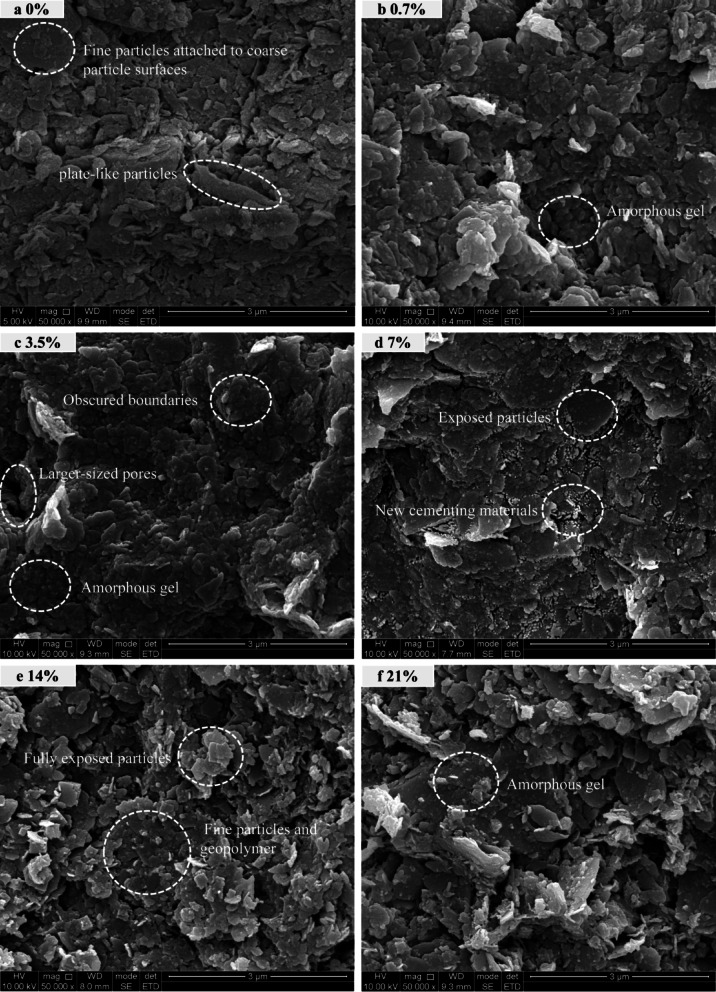
SEM images of red clay treated with alkali solutions of different concentrations at 50,000× magnification. (**a**) Plain soil (0%). (**b**) 0.7%. (**c**) 3.5%. (**d**) 7%. (**e**) 14%. (**f**) 21%.

To observe variations in particle morphology with alkali concentration, SEM images magnified 50,000 times were obtained (Fig. 7). As shown in Fig. 7, the plain soil exhibits a typical flaky particle structure, with particles interlocking with each other. Its surface is covered with abundant fine particles or cementations, resulting in a relatively rough and irregular overall morphology. At an alkali concentration of 0.7%, partial mineral dissolution occurred, leading to the dispersion of large particles into smaller ones. Amorphous gel substances adhered between particles, causing tighter interparticle connections and an increase in surface smoothness. When the contamination concentration reached 3.5%, the soil particles dispersed further, with particles enveloped by substantial amorphous gels resulting in obscured boundaries. The overall morphology exhibited significantly smooth and fine-textured characteristics, accompanied by the formation of larger-sized pores within the soil mass. As the alkali concentration increased to 7%, the amorphous gels coating the particle surfaces gradually diminished. New cementing materials generated by the ongoing alkali-soil reactions began to appear, manifested as abundant fine-grained white cementations at particle edges. These cementations bound the soil particles and filled the soil pores. When the concentration reached 14%, the amorphous gels produced by mineral dissolution almost completely disappeared. The particles became exposed, exhibiting a fragmented, randomly oriented arrangement and interlocking to form larger particles. Simultaneously, abundant fine-grained cementing materials were present on the particle surfaces and edges. When the concentration further increased to 21%, partial dissolution of the cementing materials occurred again. The particle surfaces gradually became smoother, and the white interparticle cementations decreased.

The SEM results indicate that the variations in particle morphology characteristics align well with the changes in soil strength, pore structure, and particle size characteristics. This further confirms that 3.5% and 14% are two critical threshold contamination concentrations. Among these, the 3.5% concentration sample exhibited optimal particle dispersion, the most abundant amorphous gels, and the smoothest surface features; whereas the 14% concentration sample displayed the most significant surface roughness and particle interlocking phenomena.

#### Analysis of mineral phases

**Fig. 8:**
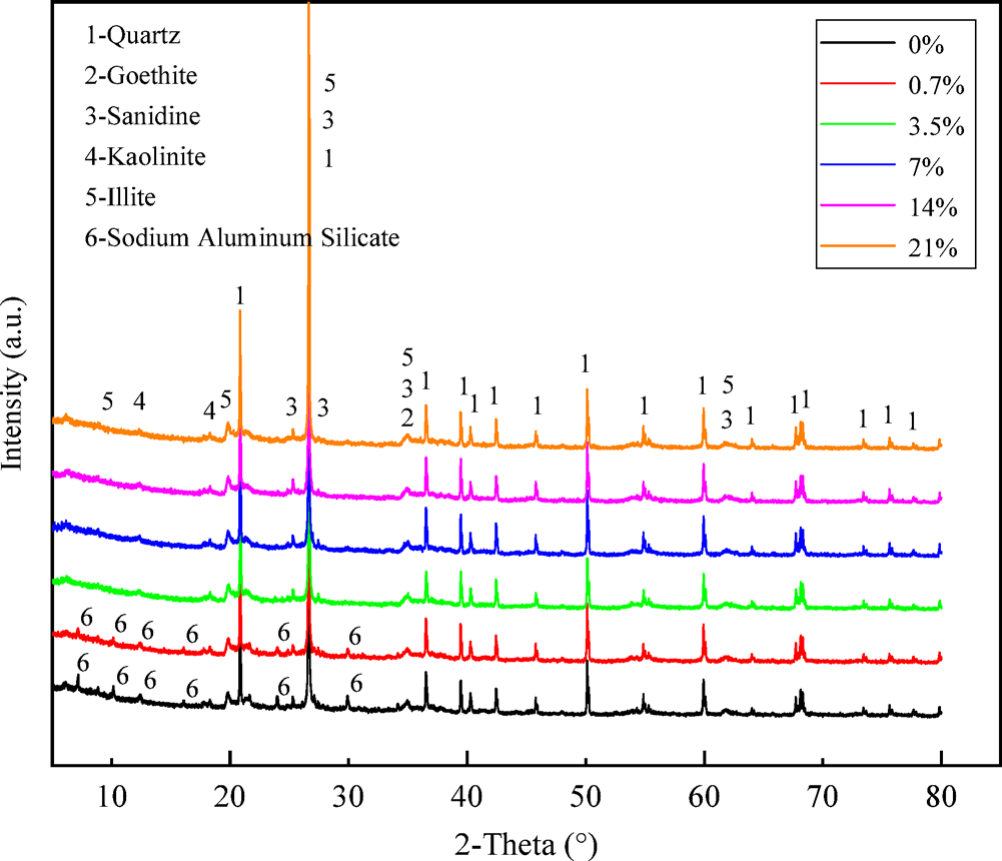
XRD pattern of alkali-contaminated red clay.

Figure 8 presents the XRD patterns of red clay subjected to alkali contamination at varying concentrations, providing direct mineralogical evidence for understanding the alkali-soil reaction pathways. The primary minerals identified in the plain soil are quartz, goethite, sanidine, kaolinite, and illite. Under contamination levels of 0.7%, 3.5%, and 7%, the XRD patterns of the soil remain nearly identical to that of the plain soil, with only slight variations in diffraction peak intensities. This indicates that at these concentrations, no new crystalline substances were formed in the red clay, or their content was too low to be detected. The alkali-soil reactions at these stages are predominantly characterized by the amorphization/dissolution of primary minerals or ion exchange. However, at the 14% contamination level, new diffraction peaks emerge in the soil sample, which are identified as sodium aluminosilicate through phase analysis. At 21% contamination, these newly formed diffraction peaks become more pronounced. The generation of these new phases strongly supports the occurrence of reconstructive cementation (geopolymerization) under high-concentration alkaline conditions^55^.

## Discussion

Red clay is rich in mineral components such as kaolinite, illite, and quartz. Under the influence of alkali solutions, these minerals undergo dissolution, disrupting the primary structure of the soil mass^56–58^, leading to the deterioration of its mechanical properties. However, numerous studies also indicate that highly alkali solutions can react with clay minerals to generate new cementitious substances^59,60^. These cementing agents can fill pores and bind particles together, thereby enhancing the mechanical strength of the soil. Additionally, metal cations in the alkali solution can replace cations on the surface of clay minerals through ion exchange, altering the thickness of the double layer on the particle surfaces and influencing the interparticle attractive forces^61^. Based on the present experimental results and integrating previous research, it is proposed that the mechanism of alkali solution action on red clay involves two competing processes simultaneously: destructive dissolution and reconstructive cementation. The dominant process varies with the concentration of the alkali solution. Among these, 3.5% and 14% represent two critical threshold concentrations.

### Destructive dissolution-dominated mechanism at low concentrations

Under low-concentration alkali solution contamination (0.7% and 3.5%), the destructive dissolution process predominates, with the dissolution effect being most pronounced at the 3.5% concentration. Initially, cation exchange causes a slight reduction in the thickness of the double layer on particle surfaces, weakening electrostatic repulsion between particles. This manifests as a modest increase in cohesion under 0.7% alkali contamination. However, when particles adsorb excessive Na⁺, the double layer thickness increases instead (the “peptization effect”), enhancing repulsive forces and causing the clay to disperse from an aggregated state into individual platelets^62^. Simultaneously, during clay mineral dissolution, the densely distributed iron oxide aggregates on their surfaces are activated and migrate, weakening the cementing effect of iron oxides on clay minerals^20^. When minerals like kaolinite dissolve, the Si-O-Si and Al-O-Al bonds within their structure break, leading to the dissolution and release of layered reactive aluminum-oxygen tetrahedra and silicon-oxygen tetrahedra from the particle surfaces. These dissolution products diffuse into interparticle fissures, forming free silicate/aluminate ions, which ultimately transform into soluble complexes or amorphous gels^63,64^. Such gels exhibit a soft texture, high plasticity, and extremely low strength. When attached to particle surfaces, they act like a lubricating film. Under natural conditions, this gel tends to shrink towards particles, forming larger voids within the soil mass. Consequently, under low-concentration (especially 3.5%) alkali contamination, red clay exhibits microstructural characteristics such as particle dispersion, reduced particle size, increased proportion of macropores, and smoothed pore walls and particle surfaces. Macroscopically, this manifests as soil softening and a significant reduction in mechanical strength.

### Reconstructive cementation-dominated mechanism at high concentrations

Under high-concentration alkali contamination (7% and 14%), the reconstructive cementation process became the dominant mechanism. This process is essentially triggered by sufficient alkali substances initiating a geopolymeric reaction, with the most significant cementation effect observed at a concentration of 14%. The free substances generated from mineral dissolution, under sustained alkali activation, react to form sodium aluminosilicate gels^60^. This gel, through continued alkali activation and curing, forms a cementitious material with a three-dimensional cross-linked network structure, uniform and porous, commonly referred to as geopolymer, alkali-activated cementitious material, or hydroceramic^65,66^. Such materials possess excellent cementing properties but poor toughness and are prone to brittle failure^67–69^. XRD evidence (Fig. 8) directly supports this process: at the 14% concentration, a new crystalline phase of sodium aluminosilicate emerged. This indicates that substantial amounts of silico-aluminous material were leached from the clay minerals and participated in the polymerization reaction. The generated sodium aluminosilicate crystalline phase is a common intermediate or secondary product in geopolymer reaction systems^70^. It should be noted that XRD is sensitive to long-range ordered crystalline phases but is less capable of detecting short-range ordered amorphous gel phases. Therefore, the absence of new crystalline peaks in the XRD pattern of red clay contaminated at 7% does not preclude the simultaneous formation of aluminosilicate gels.

These newly formed gel substances create robust “bridges” and coating layers between soil particles, connecting the originally dispersed particles into a more integrated composite framework^71^. Under shear stress, this composite framework can more effectively disperse and transfer loads^72^. Simultaneously, the gel material fills the pores between clay particles, resulting in a denser soil structure. This not only reduces the deformation of the clay under stress but also increases the effective cross-sectional area for stress transfer^73,74^. As the alkali solution concentration increases, the degree of polymerization of the geopolymer gradually rises, peaking at the 14% concentration. At this stage, the proportion of nanopores in the alkali-contaminated red clay increased to 84.37%, the average particle size significantly increased to 802.9 μm, pore walls became rougher, and mechanical strength reached its highest value. However, influenced by the inherent brittleness of the geopolymer, the reconstructed cemented framework is prone to brittle failure under shear, and its stress-strain curve exhibits a typical strain-softening pattern.

### Sustained dissolution mechanism by free alkali

This study observed a decrease in strength at the 21% concentration compared to that at 14%, a phenomenon potentially attributable to the sustained destructive effect of free NaOH. A strongly alkaline environment promotes the dissolution of clay minerals, releasing reactive components such as Si and Al, which subsequently participate in polymerization reactions to form stable cementitious phases^75^. However, when the alkali concentration is excessively high (e.g., 21%), the surplus free alkali causes continuous dissolution and erosion of clay minerals while also inhibiting the formation of gel-like substances, leading to strength degradation^76–78^. Concurrently, free OH⁻ ions persistently attack the Si-O-Al bonds within the geopolymer, reversing the stable three-dimensional network back into soluble silicate and aluminate monomers. This results in the dissolution of the aluminosilicate framework and consequent strength deterioration^79^. Therefore, the strength decline observed at the 21% concentration essentially stems from a chemical environmental imbalance induced by the “presence of excessive free alkali.”

### Limitations for engineering applications

This study has identified the short-term reinforcement potential of a 14% NaOH concentration for red clay. However, considering practical engineering applications, two core issues concerning long-term safety and performance must be rigorously evaluated. The first issue pertains to the long-term stability of the geopolymer. Generally, well-formed geopolymers with a dense three-dimensional network exhibit good chemical durability and long-term strength stability in neutral or weakly alkaline environments^40^. Nevertheless, their long-term performance is highly dependent on the chemical equilibrium of the reaction system and external environmental conditions^80^. Under sustained moisture exposure, if the geopolymer network is inadequately cross-linked or contains structural defects, hydrolysis or ion exchange may occur, leading to a gradual weakening of cementation^81,82^. More critically, as inferred from the 21% concentration case in this study, excessive free NaOH can create a persistently strong alkaline internal environment that continuously erodes the formed gel phases over the long term, resulting in a decline in cementation performance^83^. Therefore, future work must involve long-term immersion tests coupled with time-series microstructural characterization to investigate the long-term performance of alkali-stabilized clay.

The second issue concerns the environmental safety of the reinforced zone, particularly the potential corrosion risks to surrounding ecosystems and substructures posed by a prolonged high-pH environment. The presence of free NaOH elevates soil pH, a key factor governing soil properties and ecological processes^84,85^. Research indicates that high pH can inhibit soil enzyme activity, alter the solubility and chemical speciation of heavy metals, and potentially lead to groundwater contamination^86,87^. Furthermore, a persistently high-pH environment threatens the safety of engineering substructures. Concrete foundations exposed to soil containing free NaOH are susceptible to alkali-aggregate reaction (AAR) and erosion by corrosive ions, both of which compromise concrete integrity and durability^88^. Contact between free NaOH and reactive silica in concrete aggregates generates expansive alkali-silica gel^89^. When the concrete matrix cannot withstand the internal pressure exerted by the swelling gel, cracking occurs, subsequently reducing the strength, stiffness, and durability of the concrete^90^. Although AAR does not directly cause steel reinforcement corrosion, the resulting cracks provide pathways for chloride ions and other corrosive agents to penetrate, thereby accelerating reinforcement corrosion^91^. Consequently, systematic investigation into the environmental impact of the alkali solution on the reinforced zone and assessment of necessary engineering mitigation measures are imperative prior to any field application.

## Conclusions

This study, utilizing UU, MIP, LPSA, SEM, XRD, and fractal dimension analysis of pore wall roughness, leads to the following conclusions:

(1) The impact of alkali contamination on the macro-mechanical behavior and microstructural evolution of red clay exhibits significant concentration dependence. The underlying mechanism involves two competing processes: destructive dissolution and reconstructive cementation. Mechanically, both a most unfavorable concentration (3.5%) and an optimal concentration (14%) exist. Microstructural analysis confirms a strong coupling between changes in soil strength and alterations in its microstructure.

(2) Under 3.5% alkali contamination, destructive dissolution predominates. The sustained dissolution of minerals such as kaolinite significantly damages the soil structure. Microscopically, this manifests as an increased proportion of macropores, smoothed pore walls, enhanced particle dispersion, reduced particle size, and smoothed particle surfaces. This microstructural deterioration results in the minimum shear strength.

(3) Under 14% alkali contamination, reconstructive cementation dominates. Sufficient alkaline material triggers a geopolymerization reaction. The newly formed cementing agents effectively fill pores and rebind dispersed particles into aggregates. Microscopically, this is evidenced by an increased proportion of micropores, heightened pore wall roughness, and a significant increase in particle size. Macroscopically, it manifests as increased soil stiffness and peak mechanical strength. However, the inherent brittleness of the cementing material leads to pronounced strain-softening behavior during shear, with rapid post-peak strength degradation.

(4) Ultra-high concentration (21%) alkali contamination induces a reverse deterioration effect. The excessive alkali concentration causes sustained dissolution of clay minerals while simultaneously corroding the already-formed cementitious material, damaging the cementation network and partially disintegrating aggregates. This leads to reduced particle size, increased macropore content, and consequently, a decrease in shear strength. This threshold concentration effect reveals the existence of a critical concentration limit for the stability of the newly formed cementitious material.

This study clarifies the influence patterns of different alkali contamination concentrations on the mechanical properties and microstructure of red clay. It provides a theoretical basis for the prevention of alkaline corrosion in red clay foundations and offers a reference for alkali stabilization techniques (e.g., the 14% concentration significantly enhances shear strength). However, this study has certain limitations: the set concentration gradient of the alkali solution is limited and does not cover more detailed threshold intervals; meanwhile, the long-term effects of alkali solution action were not considered. If specific concentrations of alkali solution are planned for the stabilization of red clay foundations, future research should prioritize investigating the long-term stability of cementation products, especially their performance evolution laws under dynamically changing hydrochemical conditions. Before practical engineering application, a systematic assessment of their environmental safety and long-term durability should also be conducted.

## Data Availability

All data listed in the paper can be obtained by contacting doctoral student Wang Lianrui, but the availability of these data is restricted. Readers may access the data only upon the reasonable request and permission of doctoral student Wang Lianrui.
